# Inhibition of interleukin‐1β signalling promotes atherosclerotic lesion remodelling in mice with inflammatory arthritis

**DOI:** 10.1002/cti2.1206

**Published:** 2020-11-09

**Authors:** Dragana Dragoljevic, Man Kit Sam Lee, Cynthia Louis, Waled Shihata, Michael J Kraakman, Jacinta Hansen, Seth L Masters, Beatriz Y Hanaoka, Prabhakara R Nagareddy, Graeme I Lancaster, Ian P Wicks, Andrew J Murphy

**Affiliations:** ^1^ Division of Immunometabolism Baker Heart and Diabetes Institute Melbourne VIC Australia; ^2^ Department of Immunology Monash University Melbourne VIC Australia; ^3^ Inflammation Division Walter and Eliza Hall Institute of Medical Research Melbourne VIC Australia; ^4^ Department of Surgery Ohio State University Wexner Medical Center Columbus OH USA; ^5^ Rheumatology Unit Royal Melbourne Hospital Melbourne VIC Australia

**Keywords:** atherosclerosis, inflammation, interleukin 1β, monocytes, rheumatoid arthritis

## Abstract

**Objectives:**

Rheumatoid arthritis (RA), an inflammatory joint disorder, independently increases the risk of cardiovascular disease (CVD). IL‐1β contributes to both RA and CVD. We hypothesised that inhibiting IL‐1 signalling with the IL‐1R antagonist, anakinra, would dampen inflammation and promote resolution of atherosclerosis in arthritic mice.

**Methods:**

Low‐density lipoprotein receptor (Ldlr)‐deficient mice were fed a Western‐type diet for 14 weeks to develop atherosclerotic plaques. Mice were then switched to a chow diet, promoting lesion regression, and randomised to a control group or into groups where arthritis was induced by passive transfer of K/BxN arthritogenic serum. The arthritic mice were further randomised to vehicle or anakinra.

**Results:**

Arthritis impaired atherosclerotic lesion regression when cholesterol was lowered. This was associated with a higher burden of plaque macrophages, likely due to monocytosis, driven by myelopoiesis in the bone marrow and spleen. Interestingly, delayed intervention with anakinra had no effect on arthritis in these mice. However, a significant improvement in atherosclerotic plaque remodelling to a more stable phenotype was observed. This was associated with fewer circulating monocytes, caused by a reduction in splenic extramedullary myelopoiesis.

**Conclusion:**

We show that inhibiting IL‐1 signalling in arthritic mice with pre‐existing atherosclerosis promotes lesion remodelling to a more stable phenotype, that is less likely to rupture and cause ischemic events such as myocardial infarction. This suggests that IL‐1R antagonism may suppress CVD complications in patients with RA. Furthermore, inhibiting IL‐1β signalling in other patients with inflammatory diseases that also predispose to CVD may also benefit from anti‐IL‐1 therapy.

## Graphical abstract

In this study, we found that blocking the interleukin 1 (IL‐1) receptor with anakinra, an IL‐1 receptor antagonist, promoted remodelling of atherosclerotic lesions to a more stable phenotype in mice with inflammatory arthritis. This was associated with reduced myelopoiesis in the spleen and subsequently fewer circulating monocytes that are known to enter atherosclerotic plaques. Our data suggest that in conjunction with standard lipid‐lowering therapy, targeting IL‐1 signalling in cardiovascular comorbidities where IL‐1 is known to play a role may reduce the risk of a cardiovascular event.

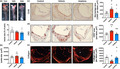

## Introduction

Patients with rheumatoid arthritis (RA), a chronic and debilitating inflammatory joint disease, have a ~ 2‐fold increased risk of atherosclerosis‐related cardiovascular disease (CVD).^1^, ^2^, ^3^ CVD is the leading cause of mortality in these individuals.^1^, ^2^ While the mechanism(s) that cause accelerated atherosclerosis in patients is unknown, it is becoming increasingly clear that it occurs independent of traditional CV risk factors and that systemic inflammation is likely to play a pivotal role.^2^, ^4^ We have previously shown that inflammatory arthritis enhances haematopoiesis and monocytosis, which directly contributes to worsened atherosclerosis in murine models of RA.^5^

Interleukin (IL)‐1β is an inflammatory cytokine that promotes inflammatory joint diseases, such as RA. *In vivo*, the biological function of IL‐1β is tightly regulated by two natural inhibitors: the decoy receptor IL‐1 type 2 receptor (IL‐1R2) and the IL‐1R antagonist (IL‐1Ra) which binds to the membrane‐bound IL‐1 type 1 receptor (IL‐1R1).^6^ Human IL‐1Ra also binds to murine IL‐1R to prevent signalling. The IL‐1Ra is essential for immune homeostasis, as mice lacking IL‐1Ra spontaneously develop destructive arthritis.^7^ Moreover, an imbalance between IL‐1β and the IL‐1Ra predisposes to RA.^8^ Analysis of synovial fluid and synovial membrane obtained from RA patients revealed increased IL‐1β expression.

The recombinant IL‐1Ra protein, anakinra, has been approved for the treatment of moderate to severe RA in patients who have failed conventional disease‐modifying anti‐rheumatic drug (DMARD) therapy. Phase III trials in people with RA showed that anakinra reduced joint inflammation and improved functional outcomes. While blocking IL‐1β in patients with RA reduces joint inflammation, its effects are less potent compared to other DMARDs.^9^, ^10^ In contrast, IL‐1 receptor antagonism more consistently reduces joint and bone damage.^9^, ^10^, ^11^

There is also an important link between IL‐1β and coronary artery disease (CAD) in RA. Ikonomidis and colleagues have shown that patients with both RA and CAD had markedly higher IL‐1β levels compared to patients with RA, but without overt signs of CAD.^12^ Interestingly, inhibiting IL‐1β in individuals with RA using anakinra improved left ventricular function and endothelial function, as well as reducing inflammation as evidenced by lower levels of CRP and IL‐6.^13^

In addition to RA, the pathogenic involvement of IL‐1β in animal models of atherosclerosis is well documented.^14^ The therapeutic value of antagonising IL‐1β was recently explored in the CANTOS trial, a study of patients at high risk of secondary myocardial infarction.^15^ The CANTOS trial targeted IL‐1β using a neutralising monoclonal antibody (canakinumab). There was a modest but significant decrease in cardiovascular events in the patients receiving canakinumab. Importantly, this was not due to reductions in circulating cholesterol levels, which remained unchanged between placebo and treatment, but rather due to reduced inflammation.

Therefore, given the importance of IL‐1β in both RA and CVD, we hypothesised that IL‐1β signalling exacerbates atherosclerosis in RA. We also reasoned that patients with CAD who also have co‐existing IL‐1‐driven diseases such as RA would stand to benefit most from anti‐IL‐1 therapy. Given that RA generally affects adults who already carry an existing atherosclerotic burden, we focused on the potential to reverse existing atherosclerosis in a pre‐clinical model of RA.

## Results

Rheumatoid arthritis is a relatively common disease of middle‐aged and older adults who undoubtedly have an existing atherosclerotic burden. Therefore, we explored the effects of blocking IL‐1β using an approved drug for RA (anakinra), in the clinically relevant setting of atherosclerotic regression. We employed the K/BxN serum‐transfer model of inflammatory arthritis as this is driven by interleukin (IL)‐1β signalling^16^, ^17^ and *Ldlr* ^−/−^ mice on a C57BL/6 background are susceptible, as we have previously shown.^5^

**Figure 1:**
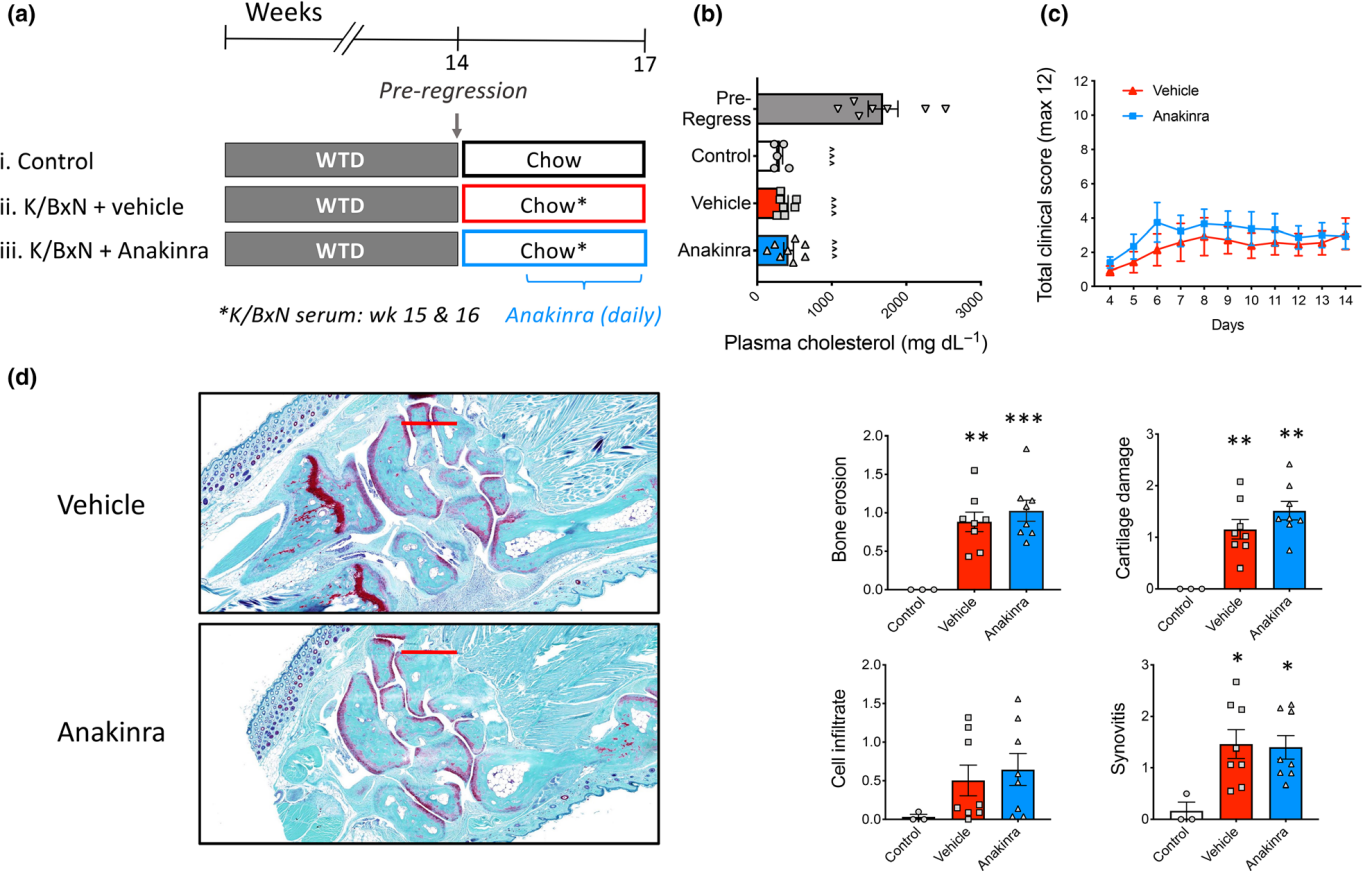
Characteristics of atherosclerotic lesion regression in inflammatory arthritis. **(a)** Experimental overview: Female *Ldlr* ^−/−^ mice were fed a western‐type diet (WTD) for 14 weeks to establish atherosclerotic lesions. Mice were then switched to a chow diet to lower plasma cholesterol levels and randomised to (i) Control, (ii) K/BxN arthritis (vehicle) and (iii) K/BxN + anakinra (anakinra). Arthritis was induced at week 15, and vehicle or anakinra was administered daily for the final 2 weeks. **(b)** Plasma cholesterol levels. **(c)** Arthritic clinical score. **(d)** Histology (scale bars = 500 mm) and analysis of the joints. One‐way ANOVA with Tukey’s post‐test, ^^^*P* < 0.001 *cf*. baseline and *P < 0.05, **P < 0.01, ***P < 0.001 *cf*. control, biological replicates *n* = 5–8 per group, **b** also included 2 technical replicates.

To mimic a clinical scenario in a pre‐clinical model, *Ldlr* ^−/−^ mice were fed a western‐type diet (WTD) for 14 weeks to promote the development of atherosclerosis. Animals were then switched to a chow diet to normalise plasma cholesterol and induce the regression of atherosclerotic lesions.^18^ One week into this regression period, mice were randomised to the following groups: (1) Control regression (control), (2) K/BxN arthritis + vehicle (Vehicle), and (3) K/BxN arthritis + anakinra (anakinra; daily for 10 days) (Figure 1a). Importantly, to recapitulate the clinical setting, anakinra was not commenced until day 4, when joint inflammation is evident in the K/BxN model. Compared to pre‐regression plasma cholesterol levels after 14wks of WTD feeding, all three groups undergoing lesion regression had markedly reduced plasma cholesterol at the conclusion of the study (Figure 1b). Importantly, there were no differences in cholesterol levels between the control, arthritic mice, or mice with arthritis receiving anakinra treatment (Figure 1b). Previous studies revealed that inhibiting IL‐1β prior to inducing arthritis prevents joint destruction.^16^, ^17^ However, in this model of delayed intervention with anakinra, we observed no improvement in arthritic clinical scores (Figure 1c). We also observe no improvement in joint histology of arthritic mice receiving anakinra, as evidenced by no change in bone erosion, cartilage damage, cell infiltrate or synovitis score compared to vehicle‐treated arthritic mice (Figure 1d).

**Figure 2:**
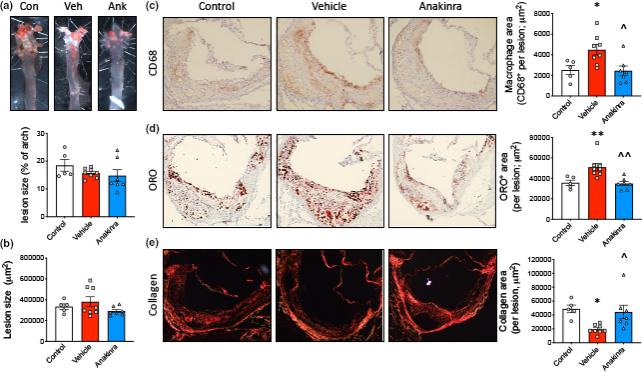
Anakinra promotes lesion remodelling in inflammatory arthritis. **(a)** Lesions in the aortic arch were quantified following Oil Red O (ORO) staining and *en face* analysis. **(b–e)** Lesions in the aortic sinus were quantified for **(b)** size, **(c)** macrophages (CD68), **(d)** lipid content (ORO) and **(e)** collagen (picrosirius red; under polarised light). One‐way ANOVA with Tukey’s post‐test, *P < 0.05, **P < 0.01 *cf*. control and ^*P* < 0.05, ^^*P* < 0.01 *cf*. vehicle, biological replicates *n* = 5–8 per group.

While there was no change in measure of joint disease, we next explored the size and composition of the atherosclerotic lesions from these mice to determine if anakinra may promote lesion regression or remodelling. First, we assessed atherosclerotic lesion size in both the aortic arch and sinus, observing no significant difference between any of the groups (Figure 2a and b). However, consistent with our previous findings,^5^ when we explored the composition of the atherosclerotic plaques, the lesions from arthritic mice displayed an unstable phenotype, evidenced by a significantly higher macrophage and lipid burden, as well as a decrease in collagen content (Figure 2c–e). Inhibiting IL‐1β signalling with anakinra resulted in a significant reduction in macrophage and lipid content, while stimulating collagen deposition in plaques of arthritic mice (Figure 2c–e). These data show that blocking IL‐1β in the setting of arthritis promotes lesion remodelling and increases plaque stability, during lesion regression when plasma cholesterol is lowered. This suggests that the persistence of atherosclerosis in inflammatory arthritis is at least in part due to systemic inflammation (Figure 1).

**Figure 3:**
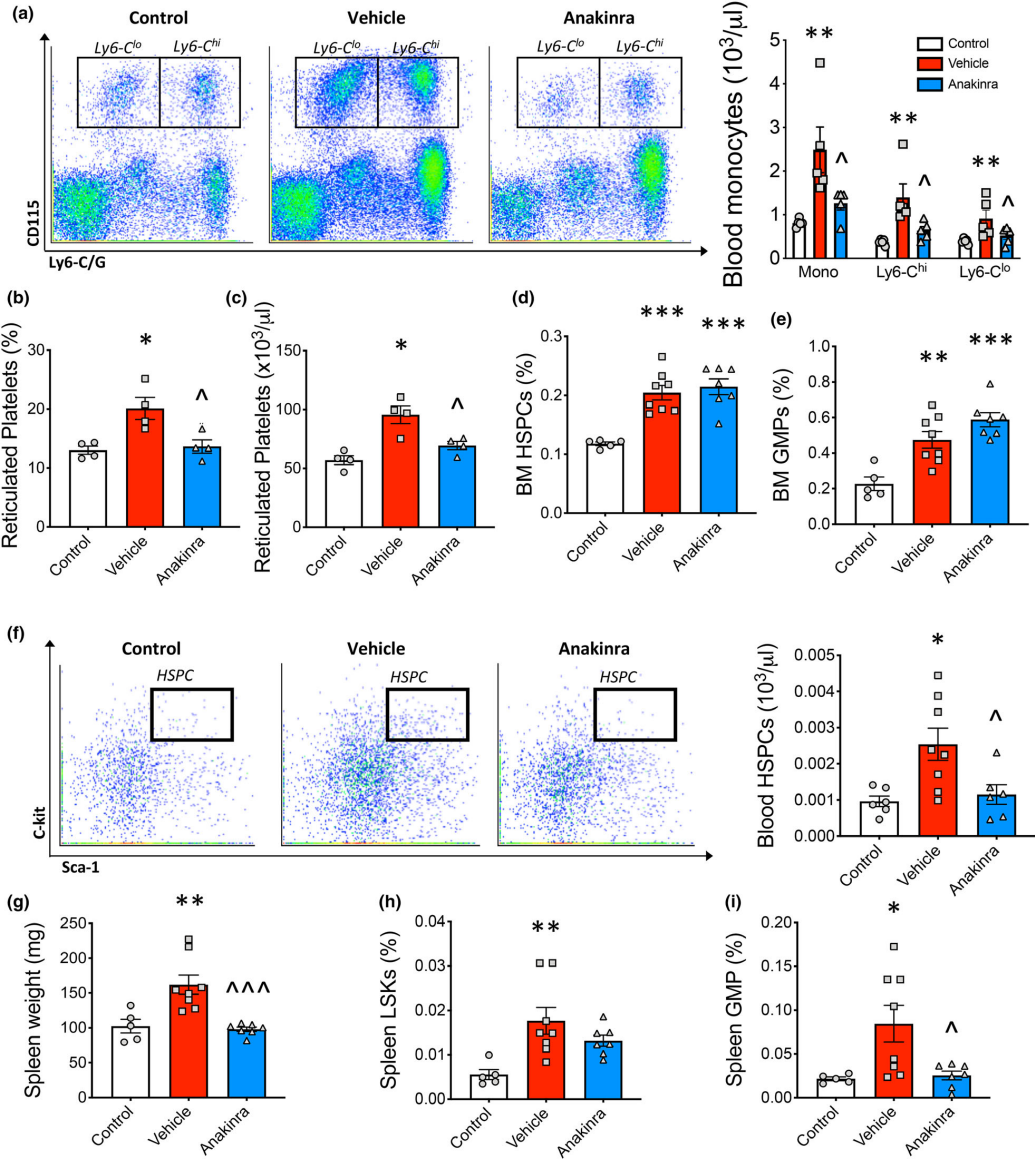
Anakinra reduces extramedullary myelopoiesis in inflammatory arthritis. **(a)** Blood monocytes, **(b, c)** reticulated platelets (% and number, *n* = 4 per group), **(d)** bone marrow (BM) HSPCs and **(e)** GMPs along with **(f)** blood HSPCs were quantified by flow cytometry. **(g)** Spleen weight. **(h)** Splenic HSPCs and **(i)** GMPs were quantified by flow cytometry. One‐way ANOVA with Tukey’s post‐test, *P < 0.05, **P < 0.01, ***P < 0.001 *cf*. control, ^*P* < 0.05, ^^^*P* < 0.05 *cf*. vehicle, biological replicates *n* = 5–8 per group.

We have previously shown that monocytosis in RA instigates increased monocyte lesion entry and, consequently, increased macrophage burden.^5^ Given that we observed a reduction in plaque macrophages with anakinra administration, we next assessed blood monocyte profiles, as lesional macrophages are known to arise predominately from circulating monocytes. Arthritis caused monocytosis in *Ldlr* ^−/−^ mice, which was reversed by anakinra treatment (Figure 3a). In a randomly selected subgroup of mice, we explored the abundance of reticulated platelets, which can also promote atherosclerosis^19^, ^20^, ^21^ and found their abundance (frequency and number) also increased in the arthritic mice, and was reduced by blocking IL‐1β (Figure 3b and c).

We previously discovered that monocytosis in RA is due to both medullary and extramedullary pathways in the spleen.^4^ First, we explored the abundance of the haematopoietic stem and progenitor cells (HSPCs) and the downstream granulocyte–macrophage progenitors (GMPs) within the bone marrow (BM) which give rise to monocytes. As expected, we observed an expansion of these bone marrow progenitor cells in arthritic mice; however, this was not reversed by anakinra (Figure 3d and e). Next, we looked for evidence of altered extramedullary myelopoiesis in the spleen. Predictably, arthritic mice displayed signs of extramedullary myelopoiesis, evidenced by mobilisation of HSPCs to the blood, and an increase in spleen size along with expanded splenic HSPCs and GMPs compared to the control. Splenic weight as well as GMP abundance was normalised in the arthritic mice treated with anakinra (Figure 3f‐i). Taken together, these results suggest that IL‐1R antagonism inhibits extramedullary myelopoiesis in RA, which is associated with a reduction in blood monocytes and an improvement in atherosclerotic lesion regression.

## Discussion

The new era of targeted anti‐inflammatory therapy with biological antagonists has allowed scientists and clinicians to begin to investigate the pathological link between systemic ‘inflammatory’ diseases and CVD. This includes the relationship between autoimmune‐mediated inflammatory diseases and accelerated CVD. A prime example of this is the CANTOS trial targeting IL‐1β as secondary prevention after a myocardial infarction.^15^ This trial reported a reduction in CV events independent of lipid levels, although the effect size was modest. This led us to hypothesise that a more targeted approach for anti‐IL‐1β therapy in a pathology where IL‐1β has a confirmed causative role. Thus, we postulated that IL‐1R antagonism may be therapeutically beneficial for the management of CVD in co‐existing inflammatory arthritis. Indeed, we found that blocking IL‐1β using the IL‐1Ra improved atherosclerotic lesion regression, as evidenced by a reduction in lipid‐laden plaque macrophages and an increase in collagen deposition in a pre‐clinical model of RA.

Blocking IL‐1β signalling in the CANTOS trial resulted in lowered risk for both primary and secondary endpoints. While this effect was independent of plasma cholesterol, the therapeutic mechanism remains unknown. Overall, IL‐1β blockade is associated with a reduction in non‐fatal and fatal myocardial infarction, stroke, hospitalisation for unstable angina and, ultimately, a reduction CV death.^22^ In keeping with this, we have shown that IL‐1R antagonism with anakinra promotes plaque remodelling towards a stable phenotype during lesion regression in an inflammatory arthritis model. This was evidenced by decreased plaque macrophage burden and increased collagen deposition. Importantly, this parallels the findings from the CANTOS trial, where improved atherosclerotic outcome occurred in the presence of equal endpoint plasma cholesterol levels.

In this study, we show that anakinra reversed monocytosis in RA, which likely contributed to reduced monocyte infiltration and a concomitant suppression of macrophage density in atherosclerotic lesions. Comparably, the reduced CV risks in the CANTOS trial were also associated with reduced circulating leucocytes in these patients.^15^ The link between monocytosis and enhanced atherosclerotic‐CVD is well documented.^18^, ^22^, ^23^, ^24^, ^25^, ^26^ Supporting this central driving mechanism, we have previously shown a direct role for increased circulating monocytes in RA to enhanced monocyte lesion entry, which, in turn, augmented plaque macrophage content and plaque instability.^5^

While it is well known that both IL‐1β and enhanced monocyte development exacerbate atherosclerosis, the role of IL‐1β in regulating myelopoiesis has not been well studied. We have previously shown that IL‐1β signalling drives enhanced monocyte production in both chronic obesity‐driven inflammation^27^ and acute ischemic events following myocardial infarction.^28^ Similarly, in the setting of acute myeloid leukaemia, which is characterised by clonal expansion of myeloid progenitors, IL‐1β was found to be required for pathogenesis.^29^ Interestingly, the CANTOS trial revealed a decrease in cancer mortality with canakinumab treatment.^15^ Pietras and colleagues have reported that sustained IL‐1β signalling can drive myeloid differentiation in HSPCs.^30^ They propose that IL‐1β underlies disordered haematopoiesis in chronic inflammatory conditions. In support of this, we show that in the setting of inflammatory arthritis, anakinra reduced excessive monocyte production by reducing extramedullary myelopoiesis. These conclusions are supported by decreased blood HSPC abundance and increased retention of HSPCs in the BM, suggesting that anakinra reduces stem cell mobilisation in RA. Anakinra also attenuated spleen enlargement, as well as splenic GMPs, further supporting the hypothesis that blocking IL‐1β interferes with extramedullary myelopoiesis in RA.

This study highlights IL‐1β as an important link in driving myelopoiesis and concomitant instability of atherosclerotic lesions in inflammatory arthritis. Antagonism of IL‐1β resulted in a reduction in circulating monocytes, associated with an improvement in plaque stability in an atherosclerotic regression model. We suggest that patients with inflammatory diseases such as RA who have co‐existing CVD, classified as a high‐risk group, will stand to benefit most from anti‐IL‐1 therapy. Our findings provide strong pre‐clinical rationale for such a clinical trial.

## Methods

### Animals

All experiments were approved either by the Alfred Medical Research Education Precinct (AMREP) animal ethics committee (E/1719/2017/B), and all mouse procedures were carried out in accordance with NHMRC guidelines. *Ldlr* ^−/−^ mice were purchased from Jackson Laboratories, and colonies were maintained at AMREP animal facilities. All mice were housed in a normal light and dark cycle and had access to food and water ad libitum. Mice were excluded from the study if they developed diet‐induced skin lesions and were humanely euthanised.

### Experimental design

Female *Ldlr* ^−/−^ mice were fed a western‐type diet (WTD; 22% fat, 0.15% cholesterol; catalogue #SF00‐219, Specialty Feeds) for 14 weeks. To induce lesion regression, the mice were then switched to a normal chow diet for 3 weeks and randomised into three groups (i. control, ii. K/BxN + vehicle, and iii. K/BxN + anakinra). After 1 week of a chow diet, inflammatory arthritis was induced using the K/BxN serum transfer model, as stated above. One day after clinical manifestations of arthritis (Day 5), anakinra was administered daily (10 mg kg^−1^; I.P; Amgen, CA, USA) until the end of the experiment. Control and control arthritic mice received vehicle (daily, saline). Treatments were administered in a blinded manner, and data analysis was also blinded. The primary outcome was blood monocyte levels.

### Induction of K/BxN serum transfer model

*Ldlr* ^−/−^ mice received 2 × intra‐peritoneal injections of K/BxN serum (100 μL), 1 week apart, to induce arthritis.

### Histology of arthritic paws

The ankle joint was evaluated histologically. Paws of mice were fixed in 10% neutral‐buffered formalin, embedded in paraffin, sectioned at 7 µm, and stained with Safranin‐O, according to standard practice. Histological analysis was performed blinded on serial joint sections for synovitis, cell influx, cartilage damage, and bone degradation. Histology scores for each parameter are as follows: 0 = normal, 1 = mild, 2 = moderate and 3 = severe.

### Atherosclerotic lesions and characteristics

Aortic atherosclerotic lesions in the aortic root were analysed from 6 μm frozen sections.

#### Lipid content

Sectioned lesions were fixed in 10% buffered formalin (4 min), washed in PBS (4 min), and dipped in 60% isopropanol before staining in 60% ORO working solution (2 h, stock solution: 1% ORO powder in isopropanol). The slides were then washed in 60% isopropanol and distilled water. Sections were stained in Mayer’s Haematoxylin (4 min), washed in tap and distilled water (3 min each) and mounted with aquatex (#108562; Merck Millipore, NJ, USA). Sections were imaged on the Olympus FSX100 microscope 4.2 × magnification, and images were analysed using Adobe Photoshop CC.

#### Macrophage abundance

Thawed sections were fixed with paraformaldehyde (4%, 20 min), washed in PBS (2 × 5 min), incubated in pre‐chilled 3% H_2_O_2_ in methanol (20 min), and then washed in PBS (2 × 5 min). Each section was blocked with normal goat serum (NGS; 10%; 30 min; #S‐1000; Vector Laboratories, CA, USA), incubated with AVIDIN blocking solution (15 min; #SP‐2001; Vector Laboratories), rinsed in PBS, and then incubated with rat anti‐mouse CD68 primary antibody (1:200; 5% NGS; 4°C; #mca1957ga; Bio‐Rad, NSW, Australia) overnight. The slides were then washed in PBS (2 × 5 min) before being incubated with the secondary antibody (1:100; 5% NGS; 30 min; #BA‐4000; Vector Laboratories). Next, the sections were washed in PBS (2 x 5 min) and incubated with ABC avidin/biotin complex (30 min; PK‐6100; Vector Laboratories) and DAB solution (#SK‐4100; Vector Laboratories). Staining was terminated with distilled water. The sections were counterstained with Mayer Haematoxylin for 15 s and rinsed in tap water before blueing in Scotts tap water and washing in tap water. Finally, slides were dehydrated in ethanol (95% 3 min, 100% 3 × 3 min), cleared in xylene (2 × 5 min) and mounted with depex (#13515; VWR International, USA). Sections were imaged on the Olympus FSX100 microscope 4.2 × magnification, and images were analysed using Adobe Photoshop CC.

#### Collagen

Sections were thawed and fixed in pre‐chilled acetone (15 min), washed in PBS (2 × 5 min), stained in 0.1% Sirius red F3BA (1 h; #365548; Sigma, USA) and then washed in 0.01 m HCl (2 min). Subsequently, the slides were then dehydrated in alcohol (95%, 5 min; 100%, 2 × 5 min), cleared in xylene (2 × 5 min) and mounted with depex (#13515; VWR International). Sections were imaged on Olympus BX61 microscope under brightfield and polarised light 4.2 × magnification, and images were analysed using Adobe Photoshop CC.

### Mouse total cholesterol

Total serum cholesterol levels were measured from plasma of mice using the Cholesterol E kit (#999‐02601; Wako Diagnostics, Osaka, Japan), as per the manufacturer’s instructions.

### Blood leucocytes

Monocytes including the Ly6‐C^hi^ and Ly6‐C^lo^ subsets were identified using flow cytometry as previously described.^27^ Blood was collected via tail bleeding into EDTA tubes, which were immediately incubated on ice. All subsequent steps were performed on ice. Red blood cells were lysed (#555899; BD Pharm Lyse; BD Biosciences, CA, USA), and WBCs were centrifuged, washed and re‐suspended in HBSS buffer (0.1% BSA w/v; 5 mm EDTA). Cells were stained with a cocktail of antibodies against CD45‐PB, Ly6‐C/G‐PerCP‐Cy5.5 (BD Biosciences) and CD115‐APC (eBioscience, CA, USA). Monocytes were identified as CD45^hi^CD115^hi^ and further subdivided into Ly6‐C^hi^ and Ly6‐C^lo^. Samples were run on the Canto II or LSR Fortessa and analysed using FlowJo.

### Blood reticulated platelets

Platelet counts were determined using the Sysmex XS‐1000i Haematology System complete blood count analysis. To identify reticulated platelets, whole was blood was stained with Thiazole Orange (#390062; Sigma) and CD41‐APC (eBioscience) for 30 min at room temperature and subsequently analysed on the LSR Fortessa to determine the percentage of reticulated platelets (CD41^+^Thiozol orange^+^). Percentage of reticulated platelets were then normalised to total platelets to determine reticulated platelet counts.

### Blood HSPC and progenitors

Blood was harvested and WBCs isolated as described above. Cells were then stained as previously described^27^. Briefly, a cocktail of antibodies to lineage committed cells (CD45R, CD19, CD11b, CD3e, TER‐119, CD2, CD8, CD4 and Ly‐6G; all FITC; eBioscience, CA, USA) and stem cell markers Sca1‐Pacific Blue and ckit‐APC‐Cy7. Haematopoietic progenitor and stem cells (HSPCs) were identified as lin^−^Sca1^+^ckit^+^. Granulocyte–macrophage progenitors were identified using antibodies to CD16/CD32 (FcγRII/III) and CD34, classified an (lin^−^Sca1^−^ckit^+^FcγRII/III^hi^CD34). Samples were run on the Canto II or LSR Fortessa and analysed using FlowJo.

## Conflict of interest

The authors declare no conflict of interest.

## Author contributions

**Dragana Dragoljevic:** Conceptualization; Data curation; Formal analysis; Investigation; Writing‐original draft; Writing‐review & editing. **Man K.S. Lee:** Conceptualization; Data curation; Formal analysis; Investigation; Methodology; Writing‐original draft; Writing‐review & editing. **Cynthia Louis:** Data curation; Formal analysis; Investigation; Methodology; Validation; Visualization; Writing‐review & editing. **Waled Shihata:** Data curation; Formal analysis; Methodology; Writing‐review & editing. **Michael J Kraakman:** Conceptualization; Data curation; Investigation; Methodology; Supervision; Writing‐review & editing. **Jacinta Hansen:** Investigation; Methodology; Validation; Visualization; Writing‐review & editing. **Seth Masters:** Conceptualization; Investigation; Methodology; Resources; Writing‐review & editing. **Beatriz Y Hanaoka:** Conceptualization; Investigation; Methodology; Writing‐review & editing. **Prabhakara R Nagareddy:** Conceptualization; Investigation; Methodology; Writing‐review & editing. **Graeme I Lancaster:** Conceptualization; Investigation; Methodology; Supervision; Writing‐review & editing. **Ian Wicks:** Conceptualization; Funding acquisition; Investigation; Methodology; Project administration; Resources; Supervision; Writing‐original draft; Writing‐review & editing. **Andrew Murphy:** Conceptualization; Funding acquisition; Investigation; Project administration; Resources; Supervision; Writing‐original draft; Writing‐review & editing.
